# Light-mediated biosynthesis of size-tuned silver nanoparticles using *Saccharomyces cerevisiae* extract

**DOI:** 10.1007/s00449-024-03060-x

**Published:** 2024-07-14

**Authors:** Lucia Colleselli, Mira Mutschlechner, Martin Spruck, Florian Albrecht, Oliver I. Strube, Pamela Vrabl, Susanne Zeilinger, Harald Schöbel

**Affiliations:** 1 Department of Biotechnology and Food Engineering, MCI - The Entrepreneurial School, Maximilianstrasse 2, 6020 Innsbruck, Austria; 2 Department of Environmental, Process and Energy Engineering, MCI - The Entrepreneurial School, Maximilianstrasse 2, 6020 Innsbruck, Austria; 3 Institute for Chemical Engineering, Universität Innsbruck, Innrain 80-82, 6020 Innsbruck, Austria; 4 Institute for Microbiology, Universität Innsbruck, Technikerstraße 25, 6020 Innsbruck, Austria

**Keywords:** Green synthesis, Optimization, Photo-processing, LED, VIS-light

## Abstract

Bio-based production of silver nanoparticles represents a sustainable alternative to commercially applied physicochemical manufacturing approaches and provides qualitatively highly valuable nanomaterials due to their narrow size dispersity, high stability and biocompatibility with broad application potentials. The intrinsic features of nanoparticles depend on size and shape, whereby the controlled synthesis is a challenging necessity. In the present study, the biosynthesis of size-tuned silver nanoparticles based on cell-free extracts of *Saccharomyces cerevisiae* DSM 1333 was investigated. Single parameter optimization strategies in phases of cultivation, extraction, and synthesis were performed to modify the nanoparticle scale and yield. Visible light was exploited as a tool in nanoparticle production. The influence of white light on the biosynthesis of silver nanoparticles was determined by using novel LED systems with the exposition of varying irradiation intensities and simultaneous performance of control experiments in the dark. Characterization of the resulting nanomaterials by spectrophotometric analysis, dynamic light scattering, scanning electron microscopy, and energy dispersive X-ray spectroscopy, revealed spherical silver nanoparticles with controlled, light-mediated size shifts in markedly increased quantities. Matching of irradiated and non-irradiated reaction mixtures mirrored the enormous functionality of photon input and the high sensitivity of the biosynthesis process. The silver nanoparticle yields increased by more than 90% with irradiation at $1.0 \pm 0.2\text{ mW }{\text{cm}}^{-2}$ and the reduction of particle dimensions was achieved with significant shifts of size-specific absorption maxima from 440 to 410 nm, corresponding to particle sizes of 130 nm and 100 nm, respectively. White light emerged as an excellent tool for nano-manufacturing with advantageous effects for modulating unique particle properties.

**Graphical abstract:**

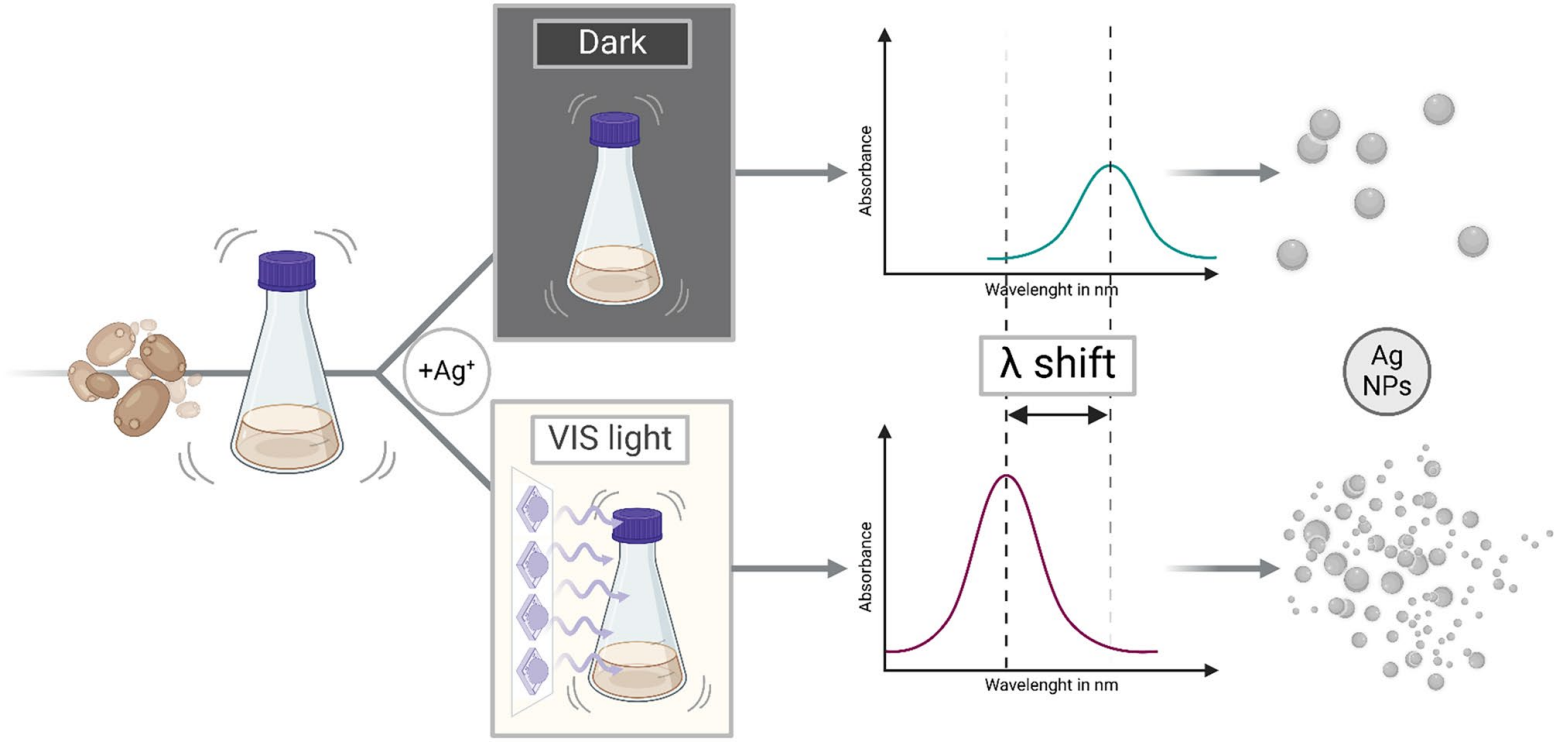

**Supplementary Information:**

The online version contains supplementary material available at 10.1007/s00449-024-03060-x.

## Introduction

Owing to their unique properties, silver nanomaterials are extensively utilized and in high demand across numerous everyday applications. The impact of silver nanoparticles (Ag NPs) on their environment is determined by their size, structure, and shape. Alongside gold, palladium, and copper, Ag NPs are among the most notable metallic nanocomponents, experiencing sharply increasing demand and being the most extensively studied due to their unique physicochemical properties [1]. They exhibit excellent antimicrobial and catalytic activities, thermal conductivity, chemical resistance, as well as magnetic and nonlinear optical performance [2]. Thus, Ag NPs find application in food technology for food processing and safety [3, 4], in biotechnological sectors and medicine for antimicrobial purposes, diagnostics and therapies [5, 6], in agriculture and environmental engineering as nano-pesticides against plant pathogens, and for wastewater remediation [7–9].

The production of Ag NPs is commercially accomplished by physical and chemical processes, which are often resource-intensive and costly [10, 11]. Alternatively, source materials of biological origin can serve as reducing agents for metal salts in NP synthesis. Bio-nanomanufacturing offers valuable nanoscale materials characterized by precise and consistent size distributions, exceptional stability, and safe methodologies, owing to their excellent biocompatibility [8, 12–14]. Bio-based synthesis of metallic NPs can be accomplished using plants or microbes. Various fungi, algae, or bacteria possess the capability to reduce metal ions in their surroundings as part of their metabolic defence mechanisms, with NP formation occurring either extra- or intracellularly within the cells [1, 15–17]. Toxic metal ions are captured through the detoxification of the cellular environment or by incorporating the metal ions into the energy metabolism as a terminal electron acceptor [16]. The primary mechanism underlying the intracellular formation of biogenic nanostructures entails the accumulation of metal ions on the cell wall via electrostatic interactions, followed by their transportation into the cell and subsequent reduction to nanoparticles mediated by enzymes. The extracellular nanoparticle formation also occurs enzymatically, where specific reducing enzymes are located in the cell wall or are secreted into the surrounding environment [1, 16, 18, 19]. In addition, several classes of bioactive macromolecules are involved in the NP-synthesis process that contribute to thermodynamic stability [18, 20]. In contrast, the stability of physicochemical generated nanomaterials is often challenging [1, 16, 18, 21]. Thus, it is necessary to produce and sustain an adequate quantity of essential biomolecules involved in the nanoparticle formation process throughout various synthesis stages, including cultivation or extraction phases. Depending on the microbial synthesis mechanism and the organisms employed as biofactories, the culture broth, resting cells, cell-free culture supernatant or aqueous cell-free extract (CFE) are usually used as reducing agents for the production of NPs [16, 22]. Using CFE offers particular merits: the effort of the downstream procedure can be minimized, since neither cell disruption, nor separation and no or only simple purification of the product is necessary. Furthermore, media components such as sugars and several anions such as Cl^–^, SO_4_^2–^ and MoO_4_^2–^ can have a strong influence on NP formation and its properties [23–25].

Microbial extracts, but also living cells, of several species of the genus *Saccharomyces* have already emerged as suitable NP production agents. Thus, successful extracellular synthesis of Ag NPs using *S. boulardii* [26], *S. uvarum* [27] as well as *S. cerevisiae* [28, 29] was described. In addition, *S. cerevisiae* proved to be a suitable cell factory to produce Au NPs [30, 31] and Se NPs [32] extracellularly as well as in an intracellular manner [33]. In most studies, yield improvements could be achieved via alterations of the process parameters such as pH, reaction temperature, or the concentration of precursor metal solution. One promising avenue in terms of increased production yields display the photoirradiation-assisted NP synthesis. Sakamoto et al. [34] and Grzelczak and Liz-Marzán [35] summarized advantages of light-induced manipulation of particle nucleation and growth and point to the resource-saving potential of light as a tool in photochemistry. By exploiting specific light characters, derived from the tight co-relation of light energy and wavelength as well as tunable intensity, photoprocessing techniques led to well-defined functionalized metallic nanomaterials with a high level of control. Especially biological nanoprocessing can benefit from the influence of light. The application of light for NP production has resulted in significantly higher efficiencies of various biochemical systems, in some cases even triggering the ability to reduce metal ions [36–39]. Ag NPs synthesized using *Cassytha filiformis* extracts exhibited highly variable dispersities, depending on the type of irradiation such as sunlight, ambient light and UV light [40]. The light-dependent biosynthesis of metal NPs based on microbial biomass often involves pigments responsible for the reduction of metal ions [38]. Thus, fucoxanthin and riboflavin have been identified as reducing agents in the light-induced formation of Ag NP using extracts of *Amphora*-46 [41] and *Pleurotus florida* [42], respectively. Nevertheless, studies regarding the selective production of Ag NPs of defined particle size and shape, which constitute major factors affecting the properties of NPs, using *S. cerevisiae* are still in their infancy and, to the best of our knowledge, no studies on the influence of visible light on the biosynthesis of Ag NPs by *S. cerevisiae* CFE have been presented so far. Therefore, the identification and optimization of an effective process cycle is imperative. However, the implementation of the NP biosynthesis using light is not trivial: it requires (i) highly standardized cultivation conditions combined with (ii) highly standardized irradiation conditions to (iii) actually ensure controlled and reproducible production.

The aim of this work was to optimize the biosynthesis of Ag NPs regarding yield and size distribution using aqueous CFE of *S. cerevisiae* DSM 1333 as reducing agent with a special focus on producing nanoscaled Ag particles of different size regimes under controlled conditions. Particular emphasis was laid on both applying moderate physical process conditions and avoiding additional chemicals to develop a green, environmentally friendly procedure. The optimization approach was divided into two parts: (i) cell-free yeast extract generation by variations of aerobic versus oxygen-limited cultivation conditions and extraction temperatures and (ii) varying parameter settings during the biosynthesis of Ag NPs with monitoring the influence of metal ion concentration, reaction time, and synthesis temperature. Furthermore, the impact of visible light on NP formation and reaction kinetics was investigated. To ensure reproducible and well-defined irradiation conditions, novel LED irradiation systems were developed and employed.

## Materials and methods

### Yeast strain

A standardized glycerol-based cryopreserved *S. cerevisiae* pure culture (DSM 1333), purchased from the German collection of microorganisms and cell cultures (DSMZ, Germany, Braunschweig), served as source for the microbial-mediated NP production. The yeast was cultured on YPD (Carl Roth, Germany) agar plates containing 1.7% agar agar (Carl Roth, Germany) at 30 °C for 72 h with subsequent storage at 4 °C.

### Initial standard procedure of Ag NP production

**Fig. 1:**
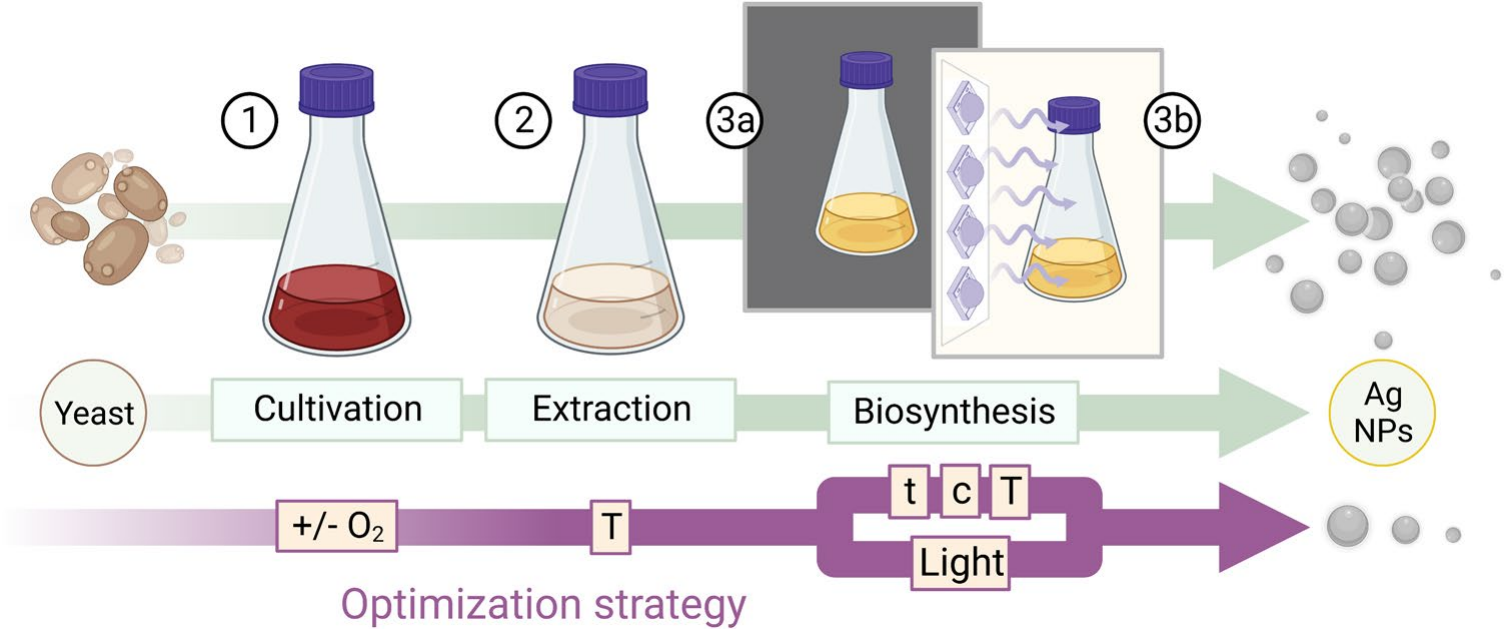
Scheme of Ag NPs biosynthesis using CFE of *Saccharomyces cerevisiae* DSM 1333 and applied single parameter optimization strategies in phases of (1) cultivation under aerobic and oxygen-limited conditions (+ /– O_2_), (2) extraction at different temperatures (T) and (3a) biosynthesis with regulations of synthesis time (t), initial Ag^+^ concentration (c), temperature (T) and (3b) VIS light irradiation

The applied workflow for biosynthesis of Ag NPs can be sectioned into three steps: (1) yeast cultivation, (2) cell-free extract preparation and (3) synthesis of NPs (Fig. 1). The initial procedure is based on methods described by Bolbanabad et al. [22] and Ammar et al. [27] with modifications.

For yeast cultivation and biomass production under aerobic conditions, approximately 1 mm^3^ of a single yeast colony was transferred from an agar plate into 50 mL YPD medium in a 100 mL Erlenmeyer flask. The culture was incubated at 30 °C and 150 rpm in the dark for 24 h. For the following experiments in triplicates and for controls, 1 mL from the previous culture was used to prepare 24 h yeast cultures in 50 mL YPD medium under the same cultivation conditions as before (Fig. 1—step 1). After 24 h cultivation period, the yeast cultures reached the late stationary growth phase. The Supplementary Information contains a microscopic image of yeast cells for reference (Supplementary Fig. S1). The yeast suspensions were adjusted to a cell density of ${\text{OD}}_{600}=5$, which corresponds to an average total cell count of $2.93\cdot {10}^{14}\text{ m}{\text{L}}^{-1}$ as determined by counting in a Thoma chamber. Yeast biomass was recovered by centrifugation at 4 °C and 5440 g for 15 min (Rotanta 460 R, Andreas Hettich GmbH & Co.KG, Germany). The obtained cell pellets were washed thrice with 20 mL ultrapure water with repeated resuspension and centrifugation to remove any remaining medium components.

To generate CFEs, the washed yeast biomass was resuspended in 50 mL of ultrapure water and extracted at 30 °C by shaking at 150 rpm (Minitron, Infors AG, Switzerland) under dark conditions for 72 h (Fig. 1—step 2). The leached yeast cells were separated at 4 °C and 5,440 g for 15 min, and the supernatant, i.e., the obtained CFE, was filtered through a 0.22 µm membrane (Rotilabo®-syringe filters, PVDF, Carl Roth GmbH + Co. KG, Germany).

For the biosynthesis of Ag NPs, precursor metal solutions were prepared by dissolving AgNO_3_ (≥ 99%, Carl Roth GmbH + Co. KG, Germany) in ultrapure water followed by filtration through a 0.22 µm membrane. Each reaction batch was prepared in triplicates, where 1 mL of AgNO_3_ solution was added to each of the CFEs in Erlenmeyer flasks with a final concentration of 0.75 mM Ag^+^ (Fig. 1—step 3a). The Ag NP biosynthesis was performed at 30 °C at a shaking rate of 100 rpm in the dark. In parallel, AgNO_3_ solution and pure CFE were treated equally as control. The reaction kinetics were monitored up to 156 h, with samples taken every 12 h initially, followed by 24-h intervals. Based on these observations, the optimization strategies were evaluated after 48 h of reaction. After visual inspection of the mixtures, spectrophotometric scans of 1 mL sample were recorded in the wavelength range of 200–800 nm at 1 nm intervals, as described below.

### Optimization strategies

#### Optimization strategies to improve NP yield and influence particle size distribution

The method optimizations were made in stages comprising cultivation, extraction, and synthesis. Single parameter evaluation studies were performed based on the above-described standard procedure. First, in addition to the described aerobic cultivation procedure (see Sect. "Initial standard procedure of Ag Np production".), *S. cerevisiae* DSM 1333 was incubated also under oxygen-limited conditions by using closed screw cap tubes (Fig. 1—step 1). During the extraction step (Fig. 1—step 2), varying thermal conditions were applied by setting temperatures to 20 °C and 40 °C, respectively. For the Ag NP synthesis (Fig. 1—step 3a), concentrations of precursor metal solutions were varied by increasing the final ion concentrations per batch from 0.75 mM to 1.0 mM and 1.5 mM Ag^+^. For further optimization, thermal treatments between 30 °C to 50 °C were performed during the synthesis (Fig. 1—step 3a).

#### Irradiation system and light-mediated Ag NP synthesis

**Fig. 2:**
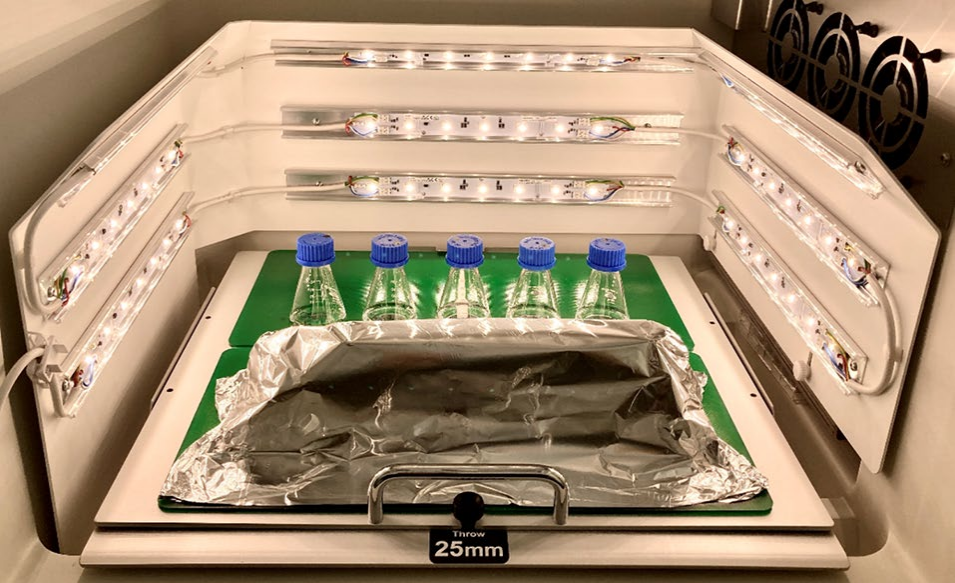
Setup of white light irradiation experiments in the incubator including an adapted LED system

To perform standardized light experiments, an irradiation apparatus specifically adapted to the used incubators (Minitron, Infors AG, Switzerland) was designed (Fig. 2). The illumination of the incubation chamber with a rotation area of $40\text{ cm}\times 40\text{ cm}$ was provided by a total of nine laterally and rearwardly centered light elements, each arraying seven LEDs emitting white light with a correlated color temperature of 3100 K (LUXEON 3535L Line, warm white, Lumileds Holding, USA). The desired irradiance was controlled via app (Casambi, planlicht®). The irradiance of visible light was determined at 555 nm by a PM100D radiometer equipped with a photodiode power sensor S120 VC (Thorlabs Inc., Newton, New Jersey, USA).

The irradiation of the reaction mixtures with white light during the NP synthesis phase was performed with an average irradiance of $1.0 \pm 0.2\text{ mW }{\text{cm}}^{-2}$ (100% intensity) and an average irradiance of $0.5 \pm 0.1\text{ mW }{\text{cm}}^{-2}$ (50% intensity) (Fig. 1 – step 3b). In parallel to the irradiated samples, a synthesis under dark conditions was performed as a control, where the samples were covered with aluminum foil (Fig. 2). Triplicates of CFEs were split into 20 mL each for irradiated and dark synthesis, each supplemented with 0.75 mM AgNO_3._ The biosynthesis was performed according to the standard procedure described above (see Sect. "Initial Standard procedure of Ag Np production") together with negative controls.

### NP characterization

The first indication of a successful Ag NP synthesis was a color change of the previously colorless transparent reaction solutions to yellowish or brown. The Ag NPs formation progress was monitored up to 156 h by recording UV/VIS absorption spectra in the range of 200–800 nm (JENWAY 7316), with 1 mL of samples withdrawn every 24 h. The morphological characteristics and elemental composition of the nanoscaled Ag particles formed after 24 h of synthesis were investigated by scanning electron microscopy (SEM) (JEOL JSM—IT200) in a magnification of 15.000 times and at an accelerating voltage of 20 kV in combination with energy-dispersive X-ray spectroscopy (EDX) (JED-2300). SEM sample preparation was performed by drop-coating carbon coated copper grids (Formvar/Carbon Supported Copper Grids, 300 mesh, Sigma-Aldrich Gmbh, Germany) with 2.5 µL of corresponding Ag NP dispersions followed by drying overnight protected from light.

The particle size distribution and the hydrodynamic diameter (Z-Average) were determined by dynamic light scattering (DLS) (Zeta-Sizer Ultra, Malvern Instruments, detector angle of 173°, 25 °C; Litesizer DLS 500, Anton Paar GmbH, detector angle of 175°, 20 °C). Three individual samples were measured for each probe and time point, with each sample subjected to three measurement repetitions (*n* = 9). Deionized water was used as dispersant. The material under investigation was colloidal silver with a refractive index of 2.58 and an absorption coefficient of 4.276.

**Fig. 3:**
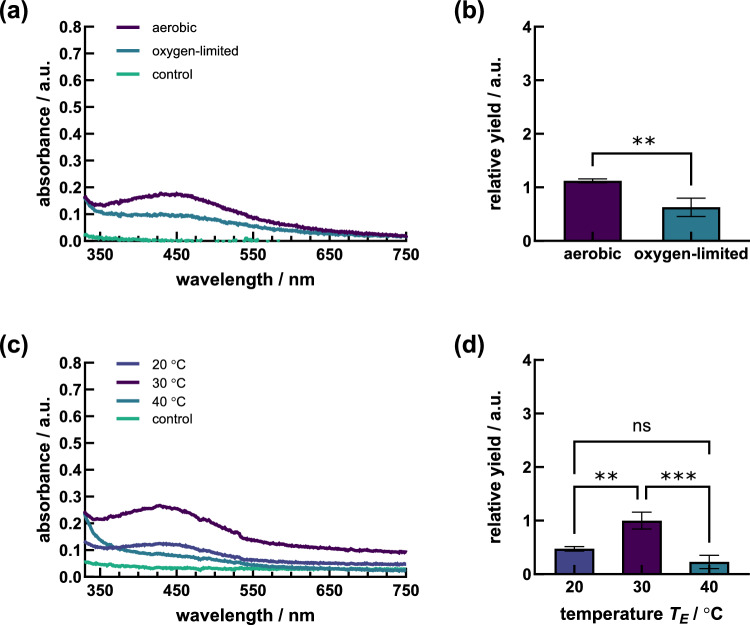
**a** Absorption spectra and **b** relative yield of Ag NPs by S. cerevisiae DSM 1333 CFEs obtained from biomass generated under either aerobic or oxygen-limited cultivation conditions **c** Absorption spectra and **d** relative yield of Ag NPs by S. cerevisiae DSM 1333 CFEs treated at different extraction temperatures of 20 °C, 30 °C and 40 °C

**Fig. 4:**
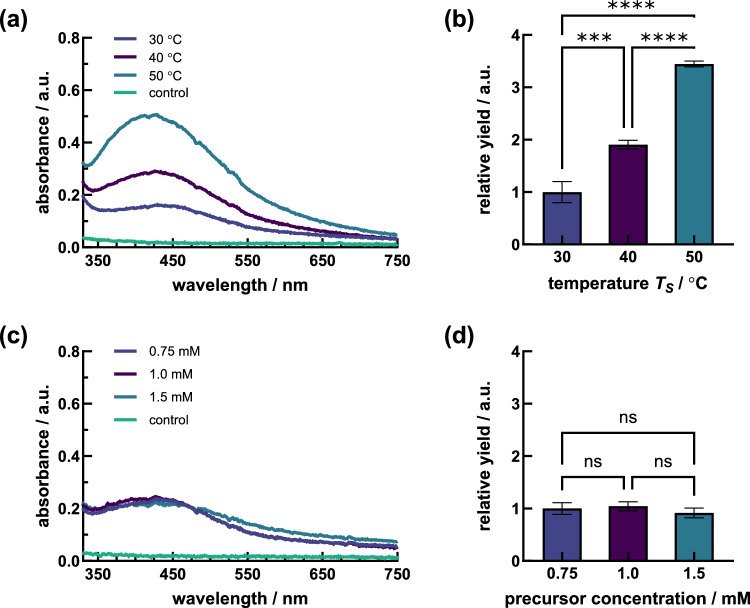
**a** Absorption spectra and **b** relative yield of Ag NPs by *S. cerevisiae* DSM 1333 CFEs at variations of synthesis temperatures of 30 °C to 40 °C and 50 °C. **c** Absorption spectra and **d** relative yield of Ag NPs by *S. cerevisiae* DSM 1333 CFEs for precursor Ag^+^ concentrations of 0.75 mM, 1.0 mM or 1.5 mM

**Fig. 5:**
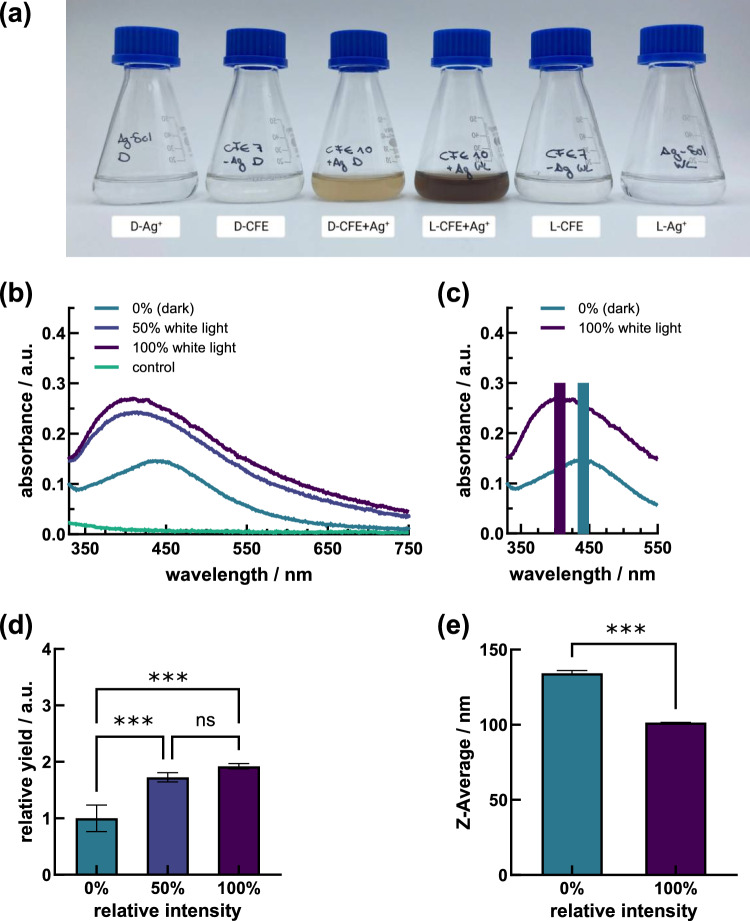
Ag NP dispersions generated by *S. cerevisiae* DSM 1333 CFEs after 48 h synthesis with resulting **a **color shifting under dark (D-CFE + Ag^+^) and irradiated conditions (L-CFE + Ag^+^), where respective colorless controls consisted of pure CFEs (D-CFE, L-CFE) and AgNO_3_ solutions (D-Ag^+^, L-Ag^+^). **b** Absorption spectra at irradiation setup of 100%, 50%, and 0% intensity, **c** peak maximum shift between non-irradiated (0%, peak maximum at 440 nm)) and irradiated (100%, peak maximum at 410 nm) conditions, **d** relative NP yield and **e** hydrodynamic diameter for peak maximum at 440 nm and 410 nm

Experiments were carried out in triplicates with resulting data averaged for evaluation. The resulting absorption spectra of the individual replicates per experiment are given in the Supplementary Information (Supplementary Fig. S3, S4, S6–S8). The absorption data of both samples and control were analyzed and processed using MATLAB (The MathWorks, Version: R2022b (9.13.0.2126072), November 17, 2022). In this process, the spectra were confined to a wavelength range from 370 to 530 nm. Subsequently, data fitting was performed with the Curve Fitting App, employing a combination of five Gaussian functions to determine the peak position of the absorption. In addition, reference values at 97% of each maximum were determined, the midpoint of which emerged as the “averaged peak”. This method served to balance potential local peaks during fitting and allowed a precise determination of peak maxima. The resulting absorption spectra of the negative controls, corresponding to pure silver nitrate solutions as well as pure CFE, were processed in parallel to the experiments and did not show any peaks in the specific detection range of Ag NPs. Ratios of detected Ag NP yields were obtained by calculating the area under the corresponding absorption peak. The area of the peak was delimited by the minimum and maximum wavelengths that defined the average peak maximum (drop of absorbance to 97% of maximum). To minimize any influence of the CFE, media residues, or silver nitrate on the absorbance, the areas of the controls were subtracted from the corresponding peak areas before calculating the relative yield. For a better overview in diagrams, controls were presented as averaged graphs. Comparisons of resulting yields are presented as normalized to the standard procedure and were used to determine the efficiency of each optimization step (Figs. 3b and d, 4b and d, 5d).

### Statistical analysis

For statistical analysis, unpaired t-test was used for two groups, and one-way ANOVA with Tukey’s multiple comparison test was used for three or more groups. Here, *corresponds to a *p*-value of *p* < 0.05, **corresponds to *p* < 0.01, ***corresponds to *p* < 0.001 and ****corresponds to *p* < 0.0001. All statistical analyses were performed using Graphpad Prism 10 (GraphPad Software LLC, USA).

## Results

### Ag NP production by *S. cerevisiae* CFE applying the initial standard procedure

The yeast strain *S. cerevisiae* DSM 1333 was screened for the potential to reduce Ag salt to Ag NPs using the aqueous CFE. Ag NP dispersions resulting from the descripted established standard synthesis method appeared after 24 h in visually detectable yellow color, with increasing intensity as biosynthesis progressed. Spectrophotometric scans indicated that Ag NPs peaked after 48 h between 420 and 440 nm (Figs. 3a and c, 4a and c, 5a and c). Microscopic imaging disclosed the spherical shape of the generated particles and EDX analysis confirmed the composition of the element Ag (Supplementary Fig. S2).

### Influence of aerobic and oxygen-limited cultivation conditions on Ag NP synthesis

CFEs obtained from aerobic and oxygen-limited yeast cultures led to significantly different Ag NP yielding (Fig. 3a and b). Ag NP suspensions based on aerobically cultivated yeasts exhibited intensive color changes during synthesis and specific resonance peaks with maxima at 440 nm, whereas those obtained from oxygen-limited cultures remained slightly colored without distinct absorption peak shaping after 47 h (Fig. 3a, Supplementary Fig. S3). The negative controls did not show any peak formation in the given range.

### Effect of extraction temperature on Ag NP formation

Aerobically cultured *S. cerevisiae* DSM 1333 biomass was extracted at 20 °C, 30 °C, and 40 °C for 72 h. The yeast CFEs extracted at 30 °C yielded the strongest yellow-brownish colored Ag NP dispersions and the most intense absorption peak formation at 426 nm (Fig. 3c, Supplementary Fig. S4). Lowering the extraction temperature to 20 °C was accompanied by strongly decreased reduction properties during Ag NP synthesis. Thermal treatment at 30 °C during the extraction also resulted in more than twofold higher Ag NP yield than at 20 °C, measured after 44.5 h of synthesis progress (Fig. 3d). Formation of Ag NPs by CFE generated at 40 °C could not be detected visually, and specific Ag NP absorption signals did not lead to peak shaping.

### Optimization of the Ag NP synthesis phase

To determine the appropriate duration of the synthesis phase, the reaction progress of Ag NP generation was monitored up to 156 h by applying the established standard conditions of 30 °C at 100 rpm and incubation in the dark. The formation of Ag NPs occurred within the first 12 h with a significant increase of specific resonance peaks and the expression of defined peak maxima during 72 h. The further course of reaction resulted in increased absorptions (Supplementary Fig. S5e), but with a concomitant loss of the tight peak shape and the tendency of macroparticle formation. The expressed peak maxima and particle size were between 36 and 84 h synthesis time without significant change at about 435 nm absorbance (Supplementary Fig. S5a) and a hydrodynamic diameter of about 130 nm (Supplementary Fig. S5c). Thus, a reasonable synthesis time for obtaining high quality Ag NP dispersions was decided to be up to 72 h.

A further optimization of the Ag NP formation was achieved by increasing the synthesis temperatures to above 30 °C. Thermal synthesis conditions of 40 °C and 50 °C during 46 h of reaction progress resulted in almost twofold and more than threefold enhanced Ag NP yields, respectively (Fig. 4b). The expressed peak maxima indicated the tendency of shifting into absorption regions of shorter wavelength with increasing synthesis temperatures with maxima at 435 nm, 426 nm and 419 nm (Fig. 4a, Supplementary Fig. S6). The controls did not change in absorbance in the observed wavelength range. Accordingly, the most intense color changes were visually observed for reaction mixtures treated at 50 °C compared with Ag NP dispersions generated at 40 °C and 30 °C. Nevertheless, the suspensions treated at 50 °C were prone to precipitation with rising synthesis duration.

As a next step, the optimal initial ion dosage was determined by performing the synthesis of Ag NPs with three different AgNO_3_ concentrations, i.e., 0.75 mM, 1.0 mM, and 1.5 mM.

The peaks in the range of characteristic Ag NP absorbance showed similar maxima (Fig. 4c, Supplementary Fig. S7) and Ag NP yields (Fig. 4d) for the initial AgNO_3_ concentrations of 0.75 mM and 1.0 mM after 46 h of the reaction progress. The resonances of Ag NPs generated with 1.5 mM initial AgNO_3_ concentration were the lowest and peak plateauing was observed. The obtained peak maxima were indicated at about 433 nm for all treatments and no peak shift between the different concentrations was obvious. The negative controls did again not show any peak formation in the given range. The coloring of the nanoparticle suspensions was more intense with increasing initial AgNO_3_ concentrations and was most intense at 1.5 mM, with turbidities of the suspensions appearing at ion concentrations of 1 mM and above after 70 h of reaction.

### Effects of light on Ag NP formation

As a further parameter, the irradiation with white light was included in the optimization of the Ag NP synthesis process, in addition to light-exclusion experiments. A visual comparison of the synthesis success revealed the intense brown color change of irradiated samples compared to yellow-colored non-irradiated samples (Fig. 5a). The negative controls remained colorless and transparent throughout the entire 156-h reaction period. Using light as an instrument for NP generation resulted in significant higher relative yields, with increases of about 70% and more than 90% at irradiation intensities of 50% and 100%, respectively, compared with Ag NPs synthesized in darkness (Fig. 5b and d).

Significant differences in the peak position were obtained when the spectra of non-irradiated versus irradiated reaction mixtures were aligned. The input of light resulted in a shift of peak localization to shorter wavelength spectral regions from maxima at about 440 nm to 410 nm, which corresponds to a hydrodynamic particle diameter of about 130 nm and 100 nm, respectively (Fig. 5c and e). The controls did not result in any peak formations in the specified range. Microscopically, the NPs generated under dark and irradiated synthesis conditions appeared in spherical shape, with a polydispersity index ≤ 0.25 for dark and irradiated conditions (Supplementary Fig. S2a and c, Table S1). The EDX analysis proved the Ag composition of the resulting particles (Supplementary Fig. S2b, d and e). Up to 84 h synthesis time, the expressed specific Ag NP absorption maxima did not show any significant changes in the localization (Supplementary Fig. S5b). As the reaction time progressed, a trend towards the appearance of larger particles or agglomeration is presumed (Supplementary Fig. S5d), accompanied by a continuous increase in relative yields (Supplementary Fig. S5f).

## Discussion

### *S. cerevisiae* DSM 1333 for Ag NP production

This study tackled the question, how the biosynthesis of Ag NPs with controlled dimensions can be accomplished in a reproducible and optimized manner using CFE of *S. cerevisiae* DSM 1333. Therefore, single parameter variations during cultivation, extraction and synthesis were performed to determine their effects on the formation and reaction kinetics of Ag NPs. The operational settings used were configured for an efficient Ag NP production along with environmentally friendly process conditions. The influences of parameter variation were monitored up to 72 h of the reaction progress. Further experiments were carried out to examine reaction times more thoroughly, encompassing a period from 12 to 156 h. These experiments aimed to offer a more comprehensive understanding of the formation kinetics of Ag NPs throughout the duration of the process (Supplementary Fig. S5). We identified no significant differences in diameter nor peak maxima between 36 and 84 h of incubation, coinciding with our preliminary data and further with previous investigations (e.g., [43, 44]), which suggest that reaction times up to 72 h are optimal. Consistent with our findings, Smiechowicz et al. [45] reported that prolonged synthesis times can influence NP generation, leading to the formation of both very small NPs and aggregates or agglomerates. Since many current methods for Ag NP synthesis are still being developed, stability issues, particularly particle aggregation, are common in NP preparations. Therefore, it is crucial to identify an optimum reaction time, as synthesized nanoparticles may tend to agglomerate with time. While there is no universal “cut-off” time, adjusting parameters like pH, temperature, and the concentration of the cell-free extract during the synthesis can help to improve stability [46]. Biological synthesis often involves biomolecules that can act as both reducing and stabilizing agents [47]. As time progresses, these biomolecules might degrade or lose their ability to stabilize the NPs effectively, leading to agglomeration. When Ag NPs aggregate, their optical properties undergo changes. UV–Visible spectroscopy emerges as a straightforward and dependable approach for assessing the stability of nanoparticle solutions. In this context, the destabilization of particles is visible in a reduction in the intensity of the original peak due to the depletion of stable NPs as well as broadening of the peak, indicating the formation of aggregates [48]. These findings align well with the observations found in the present investigation.

### Influence of aerobic and oxygen-limited cultivation conditions on Ag NP synthesis

The fungal extracellular synthesis pathway for biogenic generation of nanometric-sized materials has been used in many studies, but so far the underlying mechanisms have not been fully elucidated [49]. In this study, Ag NP synthesis efficiencies of extracts derived from oxygen-limited versus aerobically generated *S. cerevisiae* DSM 1333 biomasses were compared. The results indicated that significantly higher Ag NP intensities resulted from cultures grown under oxic conditions (Fig. 2a). To our knowledge, the synthesis of Ag NPs by *Saccharomyces* species based on oxygen-limited cultivation conditions has not yet been described. However, aerobic and oxygen-limited conditions can significantly influence the size, shape, and stability of the NPs produced and must be accounted for in the protocol refinement process using CFE of yeasts as bioagents. In this regard, differences in metabolic activity and the availability of reducing agents may lead to variations in the kinetics and mechanisms involved in NP formation. Previous studies revealed variations in both size and structure among the Ag NPs synthesized under aerobic and anaerobic conditions. In aerobic settings, the Ag NPs displayed a consistent spherical shape, while those synthesized under anaerobic conditions exhibited larger sizes and showed polydispersity [44]. Metabolic variances can further influence the accessibility of reducing agents and cofactors essential for nanoparticle synthesis. Under aerobic conditions, a conducive environment is provided for the production of essential reducing agents like NADH and NADPH, critical for metal ion reduction into NPs. Conversely, anaerobic conditions may restrict the availability of these reducing equivalents, potentially diminishing the efficiency of NP synthesis. Moreover, during yeast fermentation, the generation of organic acids such as acetic acid and lactic acid occurs concurrently with ethanol production. These acids actively contribute to the acidification of the media, resulting in a decline in pH. In this context, it is worth noting that the reduction of Ag ions is pH-dependent, impacting various aspects of Ag NP synthesis, including the rate of nucleation, particle growth, and stabilization. Qin et al. [50] investigated the influence of pH variation on the size and shape of Ag NPs, noting a trend towards smaller size and more spherical shape with increasing pH. Furthermore, synthesized Ag NPs turned out to be more stable under alkaline and neutral than under acidic pH conditions [51].

### Effects of extraction temperature on Ag NP formation

For determining the sensitivity of CFE components towards the extraction temperature, the CFE was prepared under various thermal conditions including 20 °C, 30 °C, and 40 °C. A successful Ag NPs production was achieved at an extraction temperature of 30 °C, resulting in more than a twofold higher resonance signal compared with those produced at 20 °C (Fig. 3c). However, no particle formation was detected when using CFE from yeast biomass extracted at 40 °C. In contrast to our results, Kaler et al. [26] described increased Ag NP generation using the yeast *S. boulardii* with extraction temperatures up to 40 °C, above which a decrease in yields was observed. This progression may be due to organism-dependent stabilities of proteins. Exemplarily mentioned, also plants and several microalgae belong to promising cellular machineries for the synthesis of metallic nanomaterials and the recovery of the biomolecules of interest is often achieved by preparing an infusion, i.e., at temperatures above 60 °C with boiling liquids [52–56].

### Optimization of the Ag NP synthesis phase

Since thermal regulation is one of the most critical factors for synthesizing nanomaterials as it affects particle size, size distribution, and stability [49], the abiotic parameter temperature in the synthesis phase was also investigated in the present study. The resulting Ag NP dispersions exhibited enhanced resonances with increasing synthesis temperatures from 30 °C to 40 °C and to 50 °C (Fig. 4a). A tendency of achieving different size regimes with varied synthesis temperatures was visible in a shift of the absorption maxima. According to this finding, Ag NP generation by *S. cerevisiae* DSM 1333 CFE at 30 °C and 40 °C was considered optimal, depending on the preferred particle size. The importance of organism-specific handling of NP synthesis using biogenic extracts was also discussed by Alves et al. [57], where synthesis capabilities of six different fungal extracts for Ag NP generation were evaluated at temperatures of 20 °C, 45 °C, and 90 °C. The results indicated a successful formation in an alkaline milieu and increasing temperatures, whereas only two of the candidates yielded a NP formation at pH 6 and 90 °C in an acidic milieu [57]. Conversely, a favorable Ag NP synthesis with filtrates of *Penicillium* sp. was determined by Singh et al. [14] at 25 °C, examining a temperature range of 20 °C to 45 °C.

Besides synthesis temperature variations, the optimal initial metal salt concentration was investigated. While standard procedures for fungal-mediated production of Ag NPs usually involve a concentration of 1.0 mM AgNO_3_ in reaction mixtures [26, 58, 59], we tested different initial concentrations comprising 0.75 mM, 1.0 mM, and 1.5 mM AgNO_3_. Similar absorbances of Ag NPs were detected at 0.75 mM and 1.0 mM AgNO_3_, but with tighter peak shapes at lower ion concentration (Fig. 4c). In contrast, higher ion concentrations of 1.5 mM AgNO_3_ showed both lower resonances and a plateauing of the peak. These results are consistent with observations from Singh et al. [14], who applied initial Ag salt concentrations of 0.5–2.0 mM for biosynthesis using *Penicillium* sp.. The consumption of reducing agents as well as stabilizing biomolecules and capping components, might be causative for our results. A comprehensive mechanism utilizing biological agents for NP synthesis has not yet been established due to variations in how different biological agents interact with metal ions, as well as the diverse array of biomolecules involved in NP synthesis. Organisms possessing “Silver resistance machinery” will synthesize Ag NPs only if the concentration of Ag ions remains below a specific “threshold limit.” Exceeding a certain threshold, Ag ions can induce protein precipitation, potentially leading to toxicity [26]. Thus, optimizing the initial concentration of metal precursor as well as yeast extract is pivotal for achieving desired properties and outcomes in the biosynthesis of Ag NPs. This process often requires experimental exploration to find the optimal balance among factors such as NP size, yield, stability, and toxicity. Previous studies reported that elevated concentrations of Ag salt could initially accelerate NP formation due to increased precursor availability for reduction. However, excessively high concentrations may induce aggregation or undesirable side reactions [60]. Likewise, higher concentrations of initial CFE provide more reducing agents, which can lead to faster reduction kinetics and potentially higher NP yields. However, excessive concentrations of biomolecules in the CFE can lead to increased interactions between NPs, promote more nucleation events and aggregation [60]. This can result in the formation of larger NP clusters instead of well-dispersed individual NPs, thus complicating the characterization of the synthesized NPs. Furthermore, overcrowding of the reaction mixture with biomolecules limits the availability of Ag ions for reduction resulting in a reduction of the efficiency of the NP synthesis process and yields. Lastly, using more starting material may not be cost-effective, especially if the benefits in terms of NP yield or quality do not justify the increased expense. Therefore, the concentration of Ag salt and CFE used in the biosynthesis process should be carefully optimized to minimize any potential adverse effects while still promoting NP formation. A reduced initial ion concentration is further beneficial for industrial purposes regarding raw material savings. Yet, higher metal salt concentrations also led to the successful production of Ag NPs as demonstrated by Roy et al. [61] using an extract from *S. cerevisiae* powder with 10.0 mM AgNO_3_. Another study demonstrated for a genetically modified *Pichia pastoris* strain to achieve high throughput synthesis of nanoscaled Ag components. While the living wild-type parenteral yeast strain could not resist ion concentrations above 1.0 mM, the genetically modified strain did not show a significant decline in the maximum specific growth rate up to 6 mM AgNO_3_ [62].

### Effects of light on Ag NP formation

Reports on light-mediated green synthesis of Ag NPs are sparse, yet Ag NPs exhibit prominent responsiveness to the visible light spectrum, primarily considered due to surface plasmon resonance [63], offering remarkable sensitivity and versatility for studying and manipulating light-matter interactions at the nanoscale. Notably, the wavelength of incident light appears to play a dual role: not only does it govern the rate of Ag NPs biosynthesis, but it also influences their size distribution, revealing an inverse relationship between Ag NPs size and the wavelength of visible light according to the following sequence: violet > blue > green > yellow > red > sun-light [63]. In this context, stable Ag NPs were suggested to be formed through the collaborative action of active biomolecules sourced from the yeast extract and the energy transmitted by the incident light. Biomolecules within yeast extract, such as proteins, carbohydrates, and aromatic/phenolic compounds, possess photosensitivity and may impact the synthesis of Ag NPs under white light exposure. However, the precise effects are contingent upon factors such as biomolecule concentration, light intensity and duration, and overall reaction conditions and remain to be explored in detail. Up to now, Maduraimuthu et al. [64] employed an aqueous extract of *Ulva lactuca* as a reducing agent for the formation of Ag NPs under various conditions, encompassing exposure to sunlight, normal white light (LED light), and complete darkness at room temperature for 24 h. In this context, synthesized Ag NPs under normal white light exhibited greater polydispersity compared to those synthesized under sunlight, as evidenced by a shorter SPR peak. In contrast, the reaction mixture stored in a sealed container in darkness displayed no discernible SPR band, and the color remained unchanged for up to 5 days of incubation [64]. The influence of white light even proved to be an essential factor for the generation of nanocomponents, since under dark synthesis conditions no NP formations were achieved. Mokhtari et al. [23] utilized the bacterial culture supernatant of *Klebsiella pneumoniae* as a reducing agent of Ag^+^ with rising irradiation intensity and obtained increasing Ag NPs yields, although NPs were absent under dark synthesis conditions. Similarly, Rahman et al. [65] as well as Du et al. [66] reported on the necessity of photon input for the synthesis of Ag NPs using extracellular polymeric substances of the alga *Chlamydomonas reinhardtii* and fungal cell filtrate of *Penicillium oxalicum*, respectively. Furthermore, a direct correlation was observed between light intensity and the bioproduction of Ag NPs, with reduction rates being relatively slow at lower intensities, indicative of slower photo-biochemical reaction kinetics for nucleation and growth. Conversely, at higher intensities, reduction rates were faster, resulting in smaller average sizes compared to those synthesized under low intensities [64]. These findings are consistent with our investigation observing significant effects on Ag NPs synthesis in terms of particle size and yield could be observed in our experiments (Fig. 5b–d). The achievement of reduced particle dimensions by photo-irradiation was reflected by a shift of resonances to shorter wavelength absorption regions (Fig. 5c) and was further evident from SEM images light (Supplementary Fig. S2a and c). Here, higher density of NPs and a broader size distribution became apparent by analyzing irradiated Ag NP dispersions (Supplementary Table 1). Our results are in agreement with Nuanaon et al. [67], who investigated the effects of irradiation on Ag NP formation by using *Talaromyces purpurogenus* extracellular pigment in conjunction with white light and light of defined wavelengths and further complement the work of Neethu et al. [68] who reported rapid formation of Ag NPs using *Penicillium polonicum* under ambient light conditions. The latter study attributed the faster processing time to the photosensitization of aromatic compounds. This mechanism likely involves the utilization of free electrons from these compounds by Ag ions, facilitating their reduction into Ag NPs [68]. In summary, light exerts a pivotal role in designing a rapid, cost-effective, green, and highly productive Ag NP synthesis method owing to its reduced processing duration.

Our established procedure for the yeast-based Ag NPs synthesis can be adapted to produce different metallic NPs and to investigate diverse microbial sources for their NP formation potential. The used lighting system with characterization of existing irradiation conditions enables the standardized investigation of the influence of photon input on NP production. The bioprocess coupled with the implemented technical lighting setup build a solid base for further studies to detail the correlations between defined light parameters, such as wavelength as well as irradiance, and the formation of metallic NPs [23].

## Conclusion

We succeeded in establishing an environmentally friendly standard method to produce spherical Ag NP using *S. cerevisiae* DSM 1333 CFE. The major interest of this study was in the generation of NPs with controlled size-tuning, since intrinsic features of nanomaterials are essentially underlying on their dimensions and shape.

Single process parameters were varied during cultivation, extraction, and synthesis, with optimal conditions being found at:

- Cultivation: 30 °C, dark, aerobic, 150 rpm, 24 h
- Extraction: 30 °C, dark, 150 rpm, 72 h
- Synthesis: 0.75 mM AgNO_3_, depending on preferred NP size regime 30 °C or 40 °C and dark conditions or white light irradiation, 100 rpm, up to 72 h

Thermal treatment during synthesis as well as particularly photo-irradiation led to different particle size regimes with enhanced Ag NP yields. Visible light emerged to be an excellent tool to trigger the enhanced production of Ag NPs. Parallel experiments with white light irradiation or biosynthesis of Ag nanomaterials under dark conditions differed significantly in particle quantity as well as nanoscale. The presence of light during the reduction of Ag^+^ resulted in Ag NP dispersions with higher particle density of decreased scale, indicated by shifts of size-specific absorption peaks by about 30 nm into shorter wavelength spectral regions.

The capability of *S. cerevisiae* DSM 1333 CFE to synthesize Ag NPs under dark as well as irradiated conditions compared to other bio-systems is to be emphasized, with light as a particularly valuable instrument to influence characteristics of nanoscaled products. Further studies will focus on bringing generated Ag NP in suitable form or media to ensure preservation and specific application possibilities. We aim to scale up the process with subsequent usage of Ag NPs in membrane technologies by fixation in and on different polymers with motives on exploiting antimicrobial and absorbent capacities against biofouling. Therefore, fine-tuning of Ag NP features by utilizing photo-irradiation and characterization of their qualifications requires further experiments based on the findings of this study.

## Supplementary Information

Below is the link to the electronic supplementary material.

Supplementary file1 (DOCX 2026 KB)

## Data Availability

The data underlying the findings of this study are available from the corresponding author upon reasonable request.
