# Supplementary material for: Light-mediated biosynthesis of size-tuned silver nanoparticles using Saccharomyces cerevisiae extract
Source: Bioprocess Biosyst Eng. 2024 Jul 14;47(10):1669–82. doi: 10.1007/s00449-024-03060-x (PMC11399185; doi:10.1007/s00449-024-03060-x)
Supplement: Supplementary file 1 — Supplementary file1 (DOCX 2026 KB) [file 449_2024_3060_MOESM1_ESM.docx]

# **Supplementary information**

Light-mediated biosynthesis of size-tuned silver nanoparticles using *Saccharomyces cerevisiae* extract

Lucia Colleselli ^a^, Mira Mutschlechner^a^, Martin Spruck ^b^, Florian Albrecht ^c^,
Oliver I. Strube ^c^, Pamela Vrabl ^d^, Susanne Zeilinger ^d^ and Harald Schöbel ^a,*^

a Department of Biotechnology and Food Engineering, MCI - The Entrepreneurial School, Maximilianstrasse 2, 6020 Innsbruck, Austria

b Department of Environmental, Process and Energy Engineering, MCI - The Entrepreneurial School, Maximilianstrasse 2, 6020 Innsbruck, Austria

c Department of Chemical Engineering, University of Innsbruck, Innrain 80/282, 6020 Innsbruck, Austria

d Institute of Microbiology, Universität Innsbruck, Technikerstraße 25, 6020 Innsbruck, Austria

*****Harald Schöbel**,** E-mail: [harald.schoebel@mci.edu](mailto:harald.schoebel@mci.edu)

# **Materials and Methods**

*2.2 Initial standard procedure of Ag NP production*

| 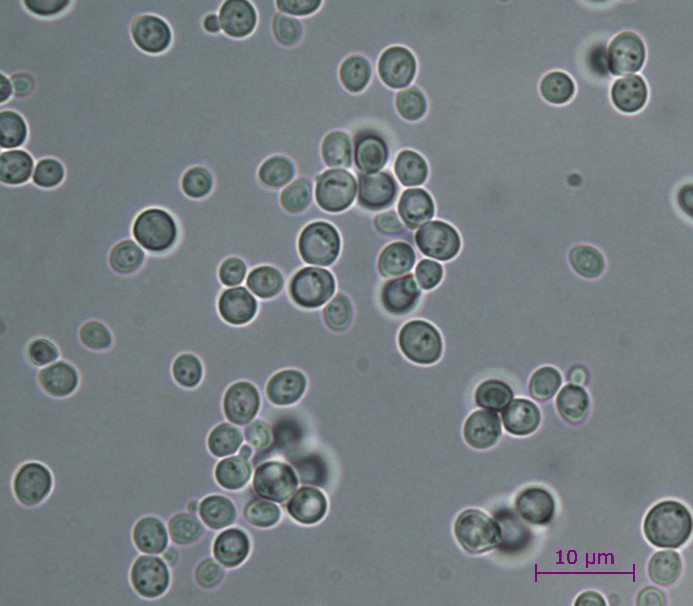 |
| --- |
| **Fig. S1** Microscope images of yeast strain *S. cerevisiae* DSM 1333 CFEs (magnification 1000x) |

# **3.Results**

*3.1 Ag NP production by S. cerevisiae CFE applying the initial standard procedure*

| 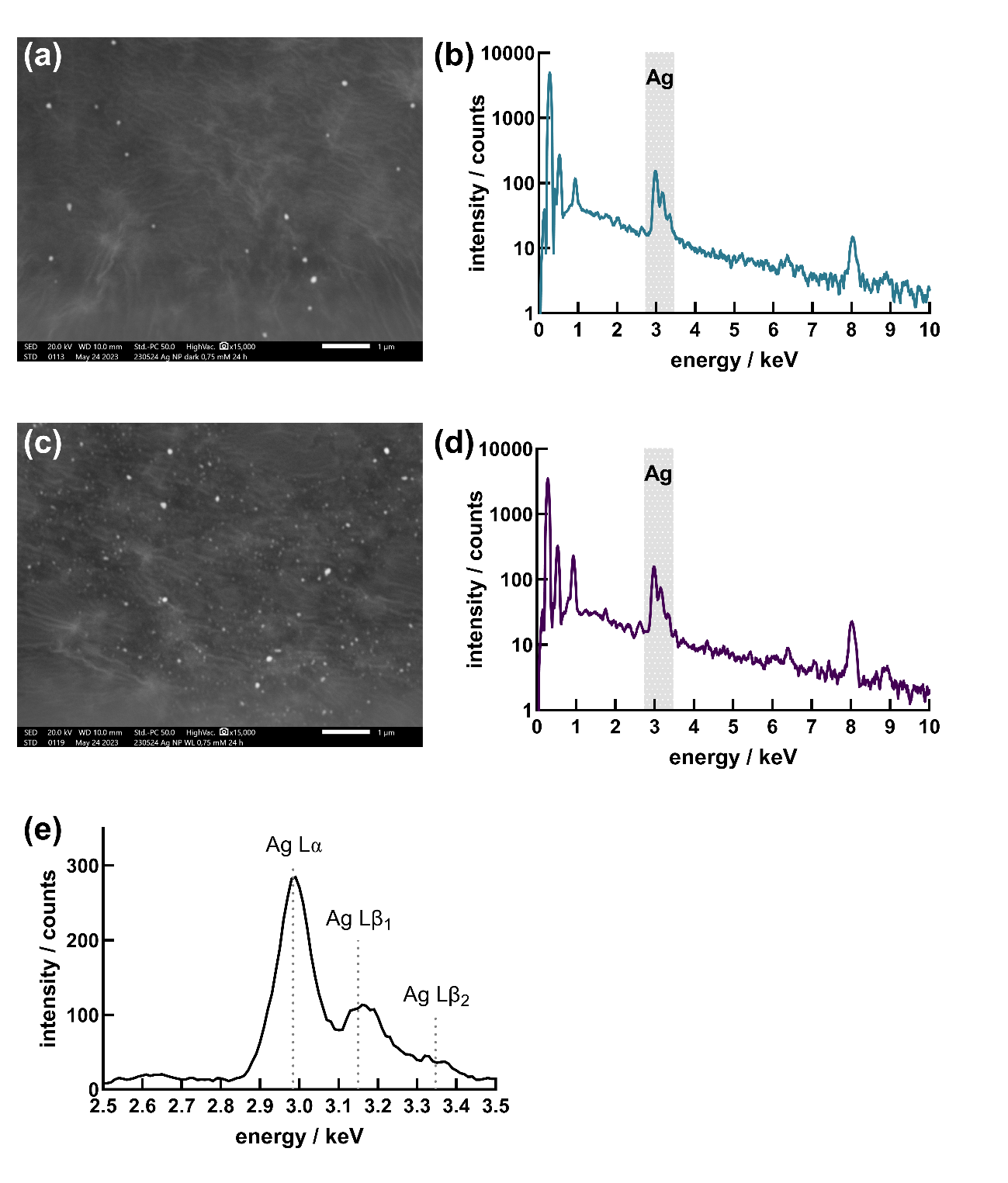 |
| --- |
| **Fig. S2** SEM images of Ag NPs (15.000× magnification, 20 kV accelerating voltage) generated by *S. cerevisiae* DSM 1333 CFEs under (**a**) dark conditions (**b**) including corresponding EDX spectra of Ag NPs and (**c**) white light irradiation at 100% intensity during synthesis (**d**) including corresponding EDX spectra of Ag NPs. (**e**) Detail of EDX spectra for the relevant energy range for the L-lines of Ag. The additional peaks in the EDX spectra are carbon (from CFE) and copper (from TEM grids). |

*3.2* *Influence of aerobic and oxygen-limited cultivation conditions on Ag NP synthesis*

| 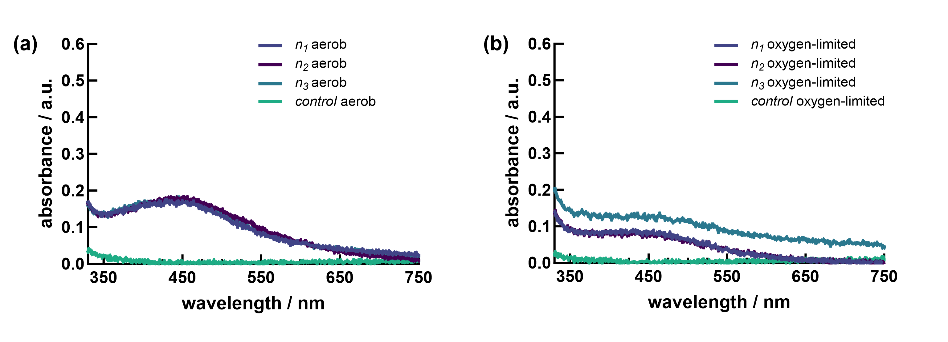 |
| --- |
| **Fig. S3** Absorption spectra of Ag NPs by *S. cerevisiae* DSM 1333 CFE in triplicates *n_1_-n_3_* and control obtained from biomass generated under either (**a**) aerobic or (**b**) oxygen-limited cultivation conditions |

*3.3. Effect of extraction temperature on Ag NP formation*

| 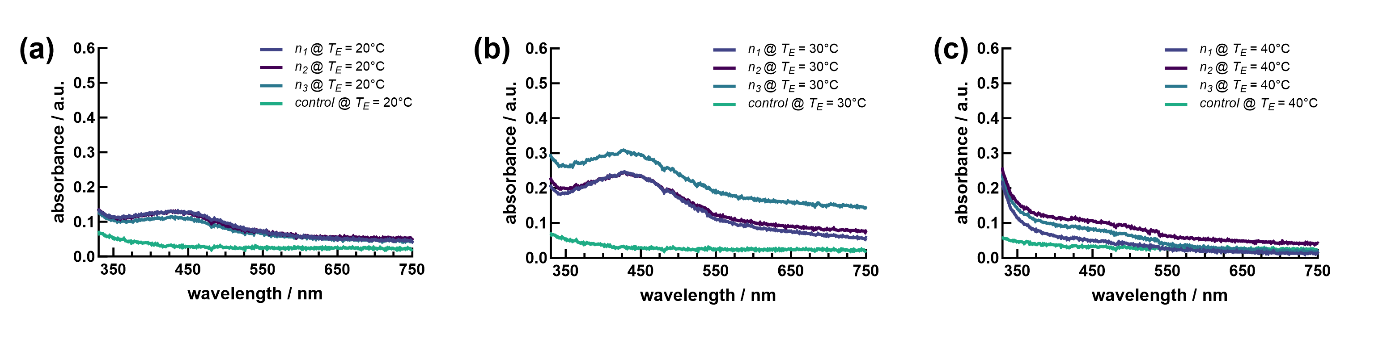 |
| --- |
| **Fig. S4** Absorption spectra of Ag NPs by *S. cerevisiae* DSM 1333 CFE in triplicates *n_1_-n_3_* and control treated at different extraction temperatures *T_E_* of (**a**) 20 °C, (**b**) 30 °C and (**c**) 40 °C |

*3.4. Optimization of the Ag NP synthesis phase*

| 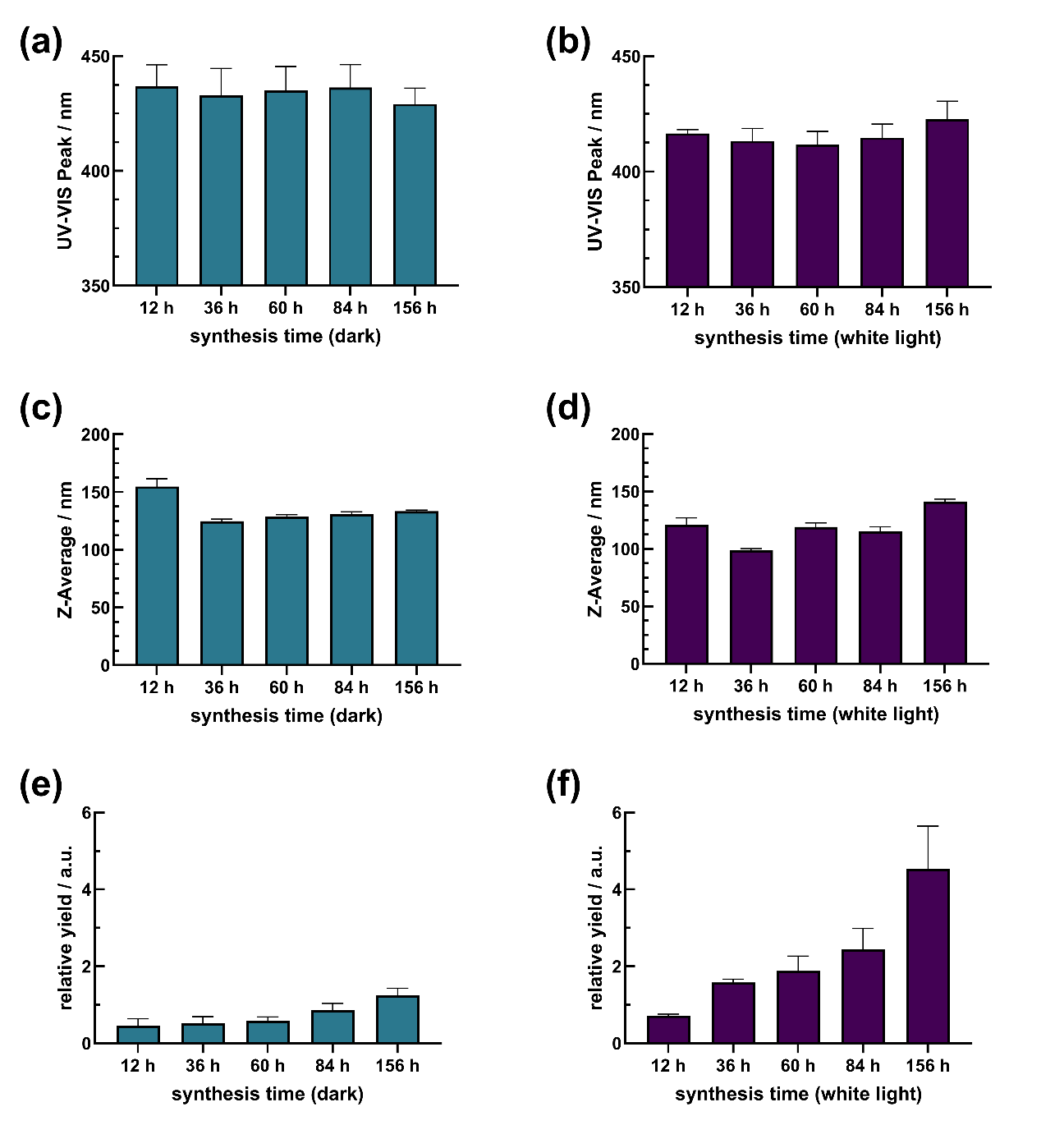 |
| --- |
| **Fig. S5** Investigation of synthesis time: (**a**) UV-VIS peak maximum (dark), (**b**) UV-VIS peak maximum (white light),  **c**) hydrodynamic diameter from DLS (dark), (**d**) hydrodynamic diameter from DLS (white light), (**e**) relative NP yield (dark), (**f**) relative NP yield (white light). |

| 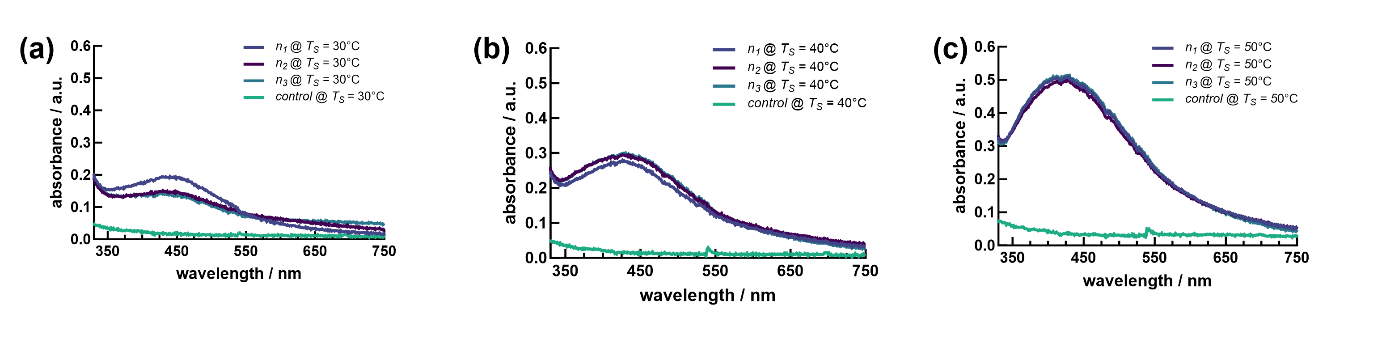 |
| --- |
| **Fig. S6** Absorption spectra of Ag NPs by *S. cerevisiae* DSM 1333 CFE in triplicates *n_1_-n_3_* and control at variations of synthesis temperatures *T_S_* of (**a**) 30 °C, (**b**) 40 °C and (**c**) 50 °C |

| 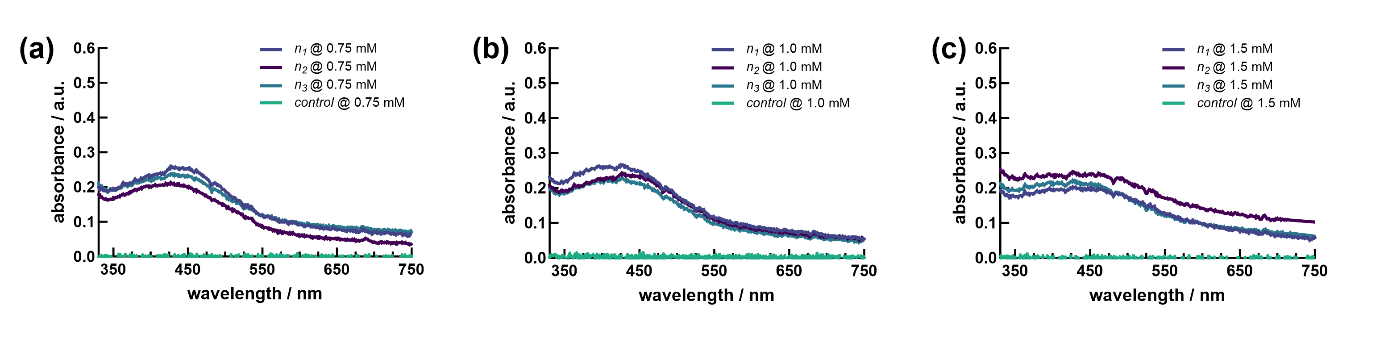 |
| --- |
| **Fig. S7** Absorption spectra of Ag NPs by *S. cerevisiae* DSM 1333 CFE in triplicates *n_1_-n_3_* and control applying precursor Ag^+^ concentrations of (**a**) 0.75 mM, (**b**) 1.0 mM or (**c**) 1.5 mM |

*3.5. Effects of light on Ag NP formation*

| 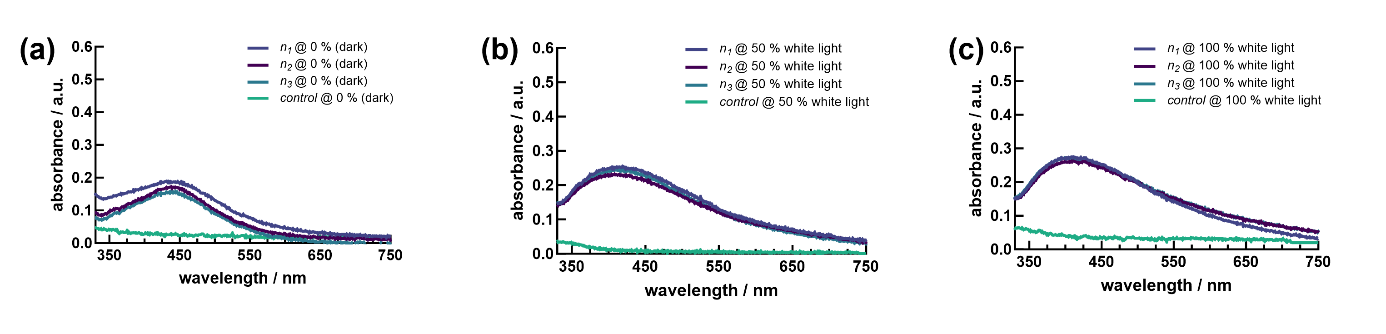 |
| --- |
| **Fig. S8** Absorption spectra of Ag NPs by *S. cerevisiae* DSM 1333 CFE in triplicates *n_1_-n_3_* and control at irradiation setup of (**a**) 0%, (**b**) 50%, and (**c**) 100% intensity |

| **Table S1:** Polydispersity Index for Ag NP synthesized with CFE from *S. cerevisiae* DSM 1333 for different synthesis times under white light irradiation and dark synthesis condidtion. | | |
| --- | --- | --- |
| synthesis time / h | polydispersity index (dark) | polydispersity index (white light) |
| 12 | $25\pm2.3$ | $24\pm3.5$ |
| 36 | $14\pm1.6$ | $22\pm1.4$ |
| 60 | $14\pm2.1$ | $17\pm2.6$ |
| 84 | $13\pm2.6$ | $15\pm3.9$ |
| 156 | $14\pm0.8$ | $22\pm3.1$ |

|  |
| --- |
|  |
